# Piercing the Pandemic Social Bubble: Disability and Social Media Use About COVID-19

**DOI:** 10.1177/00027642211003146

**Published:** 2021-11

**Authors:** Kerry Dobransky, Eszter Hargittai

**Affiliations:** 1 James Madison University, Harrisonburg, VA, USA; 2 University of Zurich, Zurich, Switzerland

**Keywords:** COVID-19, people with disabilities, social media, digital inequality, online participation

## Abstract

The COVID-19 pandemic and the ensuing stay-at-home orders caused tremendous restrictions in social contacts, which led to increasing use of the internet for daily tasks and social interactions. As prior research has established, people with disabilities (PWD) had already been using the internet for such purposes prior to the pandemic, especially for health-related content. Through a national survey administered during the first few weeks of the pandemic in the United States, we explore how people with and without disabilities used social media to exchange information and engage in activities about COVID-19. Findings reveal that PWD were more engaged with information about COVID-19 than those without disabilities, even when controlling for sociodemographics and internet experiences and skills. These differences are especially pronounced concerning more active engagement such as sharing information, interacting, and supporting others on social media. Although the content is about a health crisis in which PWD are disproportionately vulnerable, these effects largely remain when we enter controls for health status, belonging to high-risk groups for COVID-19, and personal experiences with COVID-19. Findings highlight the benefits of universal design, both for PWD specifically, and for society more broadly, as the general population ramps up use of tools long fought for and used by PWD.

The COVID-19 pandemic upended everyday life all over the world. Stay-at-home orders and business closings brought a newfound reality to many. With mobility and public activities greatly curtailed, the general population in the United States experienced a restriction of activities and social contacts unseen since the early 20th century. To deal with this disruption of everyday lives, people turned online, expanding some internet activities and learning—or being forced to learn—new ones. While having to forgo haircuts, bars, and in-person schooling, people ramped up online purchases, participated in remote work and schooling, joined video conferences, and spent time on social media participation (Hargittai & Nguyen, 2020).

In dealing with these restrictions and attempts to adapt to them, many encountered for the first time experiences that have long confronted people with disabilities (PWD). While much progress has been made in legal mandates (such as the Americans with Disabilities Act) to make aspects of public life accessible to PWD, architectural, social, and cultural barriers remain. For many PWD, these barriers result in social isolation, not unlike that experienced by the wider society during the pandemic. In order to find alternatives to an inaccessible society, many PWD have turned to the internet to find community, exchange information, and manage stigma, with social media playing a prominent role in these interactions (Kent, 2020).

Given the apparent convergence of experiences between those with and without disabilities, this paper examines the use of social media during the pandemic, focusing specifically on differences by disability status. Our research questions are as follows: Is there significant variation between those with and without disabilities in the use of social media related to COVID-19 during the pandemic? Do PWD use social media more actively than people without disabilities?

## COVID-19 and Social Inequality

As the COVID-19 pandemic swept across the United States, people from all walks of life were impacted by stay-at-home orders and the closing of schools, childcare facilities, restaurants, gyms, and retail stores. Among the widespread impacts of the pandemic has been social isolation (Banerjee & Rai, 2020; Holmes et al., 2020; Usher et al., 2020). While the most severe quarantine was imposed only on individuals who tested positive for COVID-19, wider lockdown orders forced dramatic restrictions even on those who themselves were not exposed to the virus. To adjust and find alternatives to participate in work, school, entertainment, and social interactions, many people and institutions turned online. In a Pew Research Center survey conducted in early April, 2020, over half of respondents (53%) said the internet had been “essential” for them during the pandemic, and the vast majority (87%) said it was either “important” or “essential” (Vogels, 2020). Analysis of internet use data found huge increases in online traffic, including increased interest in video chat, meeting apps and the use of social media (Koeze & Popper, 2020). A closer look, however, shows that not all experience the brunt of the pandemic equally (Robinson et al., 2020).

One disadvantaged group disproportionately impacted by the pandemic is PWD. Not only does having a disability overlap with other disadvantaged positions in society—advanced age, racial/ethnic minority status, low socioeconomic status (Taylor, 2018)—but PWD are more vulnerable to the physical and social impacts of the pandemic (Mauldin et al., 2020). Cancer, diabetes, respiratory diseases, and other chronic illnesses can be experienced as serious impairments (or disabilities) as well as put one at increased risk for severe negative outcomes of contracting COVID-19 (Centers for Disease Control and Prevention, 2020). The social costs of the pandemic also have stark effects on PWD as they disrupt healthcare access and quality, the workplace, schooling, and day-to-day interactions (Boyle et al., 2020; Mauldin et al., 2020; Morato & Pettinicchio, 2020). A survey of PWD in Wisconsin found that all respondents reported their usual care services had been disrupted by the pandemic, with over a third reporting their family had to take over these duties (Survival Coalition of Wisconsin Disability Organizations, 2020).

Much of the debate regarding whether to lift stay-at-home orders and reopen parts of society has centered on the relative risk to the vulnerable versus the needs and desires of wider society (Dobransky & Hargittai, 2020). PWD have at times lost out in such cost-benefit analyses. For instance, some medical rationing policies, which have been increasingly scrutinized during the pandemic’s taxing of medical resources, have officially deemed PWD as lower priority to receive scarce medical treatment (Bagenstos, 2020). Furthermore, while PWD have in some ways pioneered remote education and work (Gram, 2020), the abrupt move to online school and work led to problems for some PWD when issues of accessibility were not given sufficient attention (Anderson, 2020). For instance, digital learning materials might not be compatible with screen readers, and synchronous lectures might not have captioning or sign language interpreters.

Exclusion and stigmatization of PWD has a long history in the United States and elsewhere. It can be tied not only to the eugenicist drive to eliminate disability and the disabled from the population biologically but also to attempts to remove PWD from society through institutionalization or incarceration (Ben-Moshe et al., 2014). As a result (in part) of this type of marginalization, PWD have experienced increased levels of social isolation long before the onset of the pandemic. We discuss this isolation and the ways PWD have used the internet to deal with it in the next section.

## Disability, Isolation, and the Internet

PWD are more likely to live alone and less likely to work than those without disabilities (Ofcom, 2015; Taylor, 2018), thus making even incidental contact with others more difficult. For those with chronic conditions, impairments accompanying illness can lead to changes in the nature of social relationships, reducing shared activities and increasing embarrassment (Burholt et al., 2017; Bury, 1982; Charmaz, 1983; Deacon et al., 2020; Wendell, 2001). Due to anticipated stigma or experiences of being a burden on others, people may choose to self-isolate in the best of times (Charmaz, 1983; Link & Phelan, 2017) and social connections can contract (Charmaz, 1983; Deacon et al., 2020; Perry & Pescosolido, 2012).

A social model perspective highlights the importance of shifting the view from individual impairments to an inaccessible environment surrounding the person with impairments (Oliver, 2009; Shakespeare, 2014). MacDonald et al. (2018) take this approach, arguing that social isolation and loneliness are experienced most starkly in a context with inadequate resources facilitating community access, employment, and social interaction for PWD (see also Cross, 2013). That is, rather than putting the blame on PWD for their predicament of social isolation, they highlight the contextual factors that result in said isolation.

One means PWD use to deal with such isolation and loneliness is information and communication technologies (ICTs; Goggin, 2015). The internet allows PWD to form and maintain relationships and community in ways that may not be possible offline. Going online, PWD have more control over whether and how to disclose disabilities that might be readily apparent in face-to-face interaction, thus managing stigma (Saltes, 2013; Soderstrom, 2009; Tsatsou, 2020). At other times, PWD can put their impairments or health conditions in the forefront of interactions online, using ICTs to join communities of others who share their condition (Goggin & Noonan, 2006). In such forums, people can engage in peer support, mutual aid, and advocacy, challenging dominant cultural conceptions of themselves and their impairments, and offering alternatives (Barker, 2008; Chadwick & Fullwood, 2017; Ellcessor, 2016; Obst & Stafurik, 2010; Trevisan, 2017). Dobransky and Hargittai (2016) found that, controlling for sociodemographics, internet experiences, and internet skills, PWD exceed those without disabilities in some active online engagement such as sharing their own content, submitting reviews of products and services, and posting to blogs. Additionally, PWD have consistently shown more interest in health information online than those without disabilities (Andreassen et al., 2007; Dobransky & Hargittai, 2006; Fox, 2007).

Social media increasingly play a central role in interactions online and have been shown to have played an important role during the COVID-19 pandemic in particular (Nguyen et al.,2021). However, using social media to one’s benefit requires that such platforms be accessible for people of different backgrounds—including PWD – and there is a history of accessibility problems in social media. Boudreau (2012) evaluated five common social media platforms in 2012, testing the accessibility of eight different components including keyboard shortcuts, color contrasts, and alt text for images. No platform scored over 33%, which was LinkedIn’s score. Facebook scored 10%, while Twitter scored zero, meaning that it met none of the accessibility criteria. Such problems led PWD to develop their own workarounds, such as Accessible YouTube, Easy Chirp, and You Describe to make the platforms accessible (Hollier, 2017; Kent, 2020). Since then, things have improved. As Hollier (2017) explains, Twitter, Facebook, Google, and YouTube have all devoted much more attention to issues of accessibility—with Facebook partnering with the American Federation for the Blind. Barriers nonetheless remain. For instance, as we write this, Twitter is testing a feature that records a person’s voice and posts the recording as an (audio) tweet (Twitter Support, 2020a). Immediately after Twitter made this new option public, commenters raised questions about the accessibility of the feature for those with hearing impairments. Twitter responded with the disclaimer that “this is an early version of the feature,” but that they were “exploring ways” to make the feature accessible to all (Twitter Support, 2020b). Overall, however, social media are much more accessible than in the past, making it easier for PWD to take advantage of this form of online interaction.

PWD are not a unitary population, and the internet does not pose uniform challenges or benefits for people with all types of impairments (Dobransky & Hargittai, 2006, 2016). For instance, people with communicative disabilities were shown to be more concerned about their internet access early in the pandemic than those with other types of disabilities (Dobransky & Hargittai, 2020).

Given existing experiences dealing with social isolation through online options, PWD may be a few steps ahead of those without disabilities who confronted isolation for the first time due to the pandemic. Has the COVID-19 crisis simply led to a leveling of differences between those with disabilities and those without regarding isolation and internet use? In what follows, we examine social media use in the context of the COVID-19 pandemic, comparing people with a range of disabilities to those without. After presenting some distinctions among people with different types of disabilities and comparing them to those without in their general experience of the pandemic, we compare these groups’ social media uses, focusing on more active uses versus more passive uses.

## Method

We administered a survey to study people’s experiences of the pandemic, especially interested in their digital media uses during the initial weeks of lockdown (Hargittai et al.,2020).

### Data Collection

We collected data from American adults aged 18+ years a few weeks into lockdown measures across the United States: April 4-8, 2020. We contracted with the Cint online survey firm to reach a diverse sample. Research in the past decade has shown that telephone and careful opt-in online survey approaches are comparable (Ansolabehere & Schaffner, 2014). Cint uses a double opt-in panel and has a respondent pool of over 15 million people in the United States. We quota sampled on age, gender, education, and region to match U.S. Census figures. Those sociodemographic factors are often related to internet use and so we wanted to make sure our sample varied on those characteristics (Hargittai & Hsieh, 2013). Respondents come from all fifty U.S. states plus Washington, DC. At the beginning of our data collection, the United States had over 300,000 confirmed COVID-19 cases and there had been 8,360 deaths (Wikipedia, 2020). We implemented attention-verification questions (Berinsky et al., 2014) and removed cases that failed on more than one, which constituted 4.6% of the 1,441 original respondents. Our analyses are based on the 1,374 valid cases.

### Measures: Independent variables

#### Disability Status

To assess people’s disability status, we asked the following question, based the U.S. Census Bureau’s Current Population Survey (Ward et al, 2017): “Do you have any of the following long-lasting conditions? Check all that apply.” These were the options people could check off: Blindness or severe vision impairment even with glasses or contact lenses; Deafness or a severe hearing impairment even with a hearing aid; Serious difficulty having your speech understood; Serious difficulty walking or climbing stairs; Serious difficulty dressing or bathing; Serious difficulty typing on a traditional computer keyboard; Serious difficulty concentrating, remembering, or making decisions; and Serious difficulty going outside the house alone. Following the U.S. Census Bureau (Brault, 2012), we grouped the first three into communicative disabilities (such people make up 6% of the sample) and the next three into physical disabilities (8%). The second-to-last we refer to as cognitive impairments (5%), the last condition as difficulty going outside (3%). We have dummy variables for each of these types of disabilities. Additionally, to gauge the collective experience of PWD, we created a dummy variable for having any disability (16%). We included a dummy variable for having multiple disabilities (7%) to capture those dealing with more significant impairment.

#### Sociodemographics

We measured age by asking for respondents’ birth year and subtracted that from 2020. The age range is 18 to 82 with an average age of 46 years. Gender options were male, female, and other (one respondent), which we recoded into a female gender category (1 vs. 0 for all others; 54% female). We measured education level by asking for respondents’ highest level of school completed with six options, which we recoded into three: high school degree or less (49%), some college (21%), and college degree or more (29%). We asked about household income through 13 categories ranging from less than $10,000 to $200,000 or more, which we recoded to midpoint values to create a continuous variable (*x* = $59,104; *SD* = $52,157). We log this measure in the regression models.

Similar to the U.S. Census, we asked respondents separately about their ethnicity and race as is done on the U.S. Census form. First, we asked: “Are you of Hispanic or Latino descent?” with yes/no answer options. Second, we asked: “Please check one or more categories below to indicate what race or races you consider yourself to be.” with the following answer options: White (65%), Hispanic (15%), Black/African American (13%), Asian (5%), American Indian or Alaska Native, Native Hawaiian or Pacific Islander (2% for these two categories), Other, please specify. When possible, we recategorized those who chose “Other” based on the information they provided (e.g., 27 of the 45 indicated Hispanic/Latinx origin).

#### Internet Experiences and Skills

People’s experiences with using the internet may influence their active engagement online so we control for frequency of use, autonomy of use, and internet skills in the analyses. We asked people, separately for an average weekday and average Saturday/Sunday, how often they “use the internet, either on a computer, tablet, or phone.” with the following options: almost constantly, several times a day, about once a day, several times a week, less often. If on either weekdays or on weekends the respondent indicated using the internet once a day or less, we coded them as a less frequent user (10%). We also asked people what devices they “have available at home to access the internet” with mobile, tablet, laptop or desktop computer, smart TV, and gaming device as the options. We created a dummy variable for people who *only* have home internet access on a mobile device (7%) because this type of access allows more limited online engagement (Napoli & Obar, 2014). We employ a widely used internet skills measure (Hargittai & Hsieh, 2012) in which respondents rank their level of understanding of six internet-related terms (e.g., pdf, wiki) on a 1- to 5-point scale. We averaged these for the skills measure (Cronbach’s α = .90). Participants’ skills are varied with observed values ranging from 1-5, *x* = 3.3, *SD* = 1.1.

#### Health-Related Factors

People who belong to the medically high-risk category for COVID-19, those who live in a household with medical workers, and those who know others with the disease (or who themselves have been diagnosed with it) may be predisposed to be actively involved in information-seeking and interactions regarding COVID-19 because of their own increased risk. We asked people whether they have various medical conditions (e.g., high blood pressure, cardiovascular disease, cancer) that constitute high-risk and created a dummy variable for this (= 1 if yes; 37%). We also asked the following: “Do you or anyone in your household currently work at a healthcare facility, or visit a healthcare facility for work reasons, where Coronavirus (COVID-19) patients are cared for?” (12%) as well as whether they know of others who have tested positive (“Do you know any people who have been diagnosed with Coronavirus (COVID-19)?” (17%) or whether they themselves have tested positive (2%) and created another dummy variable (= 1 for a yes to any of these questions about COVID-19; 26%). We also have a measure of people’s general health status in response to the question: “In general, how is your health?” with the following answer options: poor, fair, good, very good, excellent. We created a dummy for those who indicated poor or fair (= 1, 15%). We include this in the analyses, because, like those with COVID-19 experiences above, they may be more likely to engage with COVID-19-related content online.

### Measures: Dependent Variables

To measure people’s engagement on social media related to COVID-19, we asked about the types of content they saw, shared, and engaged with actively related to the virus on such platforms. Before asking about these specifically, we established who uses certain social media platforms in the first place by asking: “How often, if ever, do you use the following sites and services?” with sites listed in randomized order. Facebook was the most popular (83% reported using it), followed by Instagram (52%), Twitter (44%), Snapchat (34%), TikTok (24%), Reddit (26%), and WhatsApp (23%). Based on the literature (Pew Research Center, 2019), we anticipated that the first three would be the most popular among U.S. respondents so we restricted subsequent questions about activities to those three to conserve survey space and since some minimal level of uptake is necessary for meaningful comparisons.

To assess social media use about COVID-19, we first asked about the types of content people were seeing on social media with the following: “Have you seen the following types of information about Coronavirus on the social media platforms listed below?” screened for the platforms they had indicated using in response to the previous question. The activities that people could check were as follows, verbatim: Tips on how to avoid getting infected; Information about symptoms; Numbers or charts about its spread; Government rules about what people are allowed to do; Religious sentiments and teachings related to it; Humor, jokes, funny content related to it; Gratitude expressed toward health care workers; and News coverage about it. From these, we created a dummy variable signaling seeing anything about COVID-19 (yes to any of the items on the above list on any of the platforms included) and a summary variable that counts up the number of types of information seen on any of the platforms. These are our measures of passive social media use on the topic of COVID-19 since they concern seeing content rather than creating it.

To assess whether people were also sharing content about COVID-19 in addition to seeing such content, we asked the following: “Have you shared the following types of information about Coronavirus on the social media platforms listed below?” (The platforms are again the ones from among Facebook, Instagram, and Twitter that they had indicated using earlier on the survey.) These were the types of information as listed verbatim on the survey: Tips on how to avoid getting infected; Information about symptoms; Numbers or charts about its spread; Government rules about what people are allowed to do; Religious sentiments and teachings related to it; Humor, jokes, funny content related to it; Gratitude expressed toward health care workers; and News coverage about it. As we did with questions regarding seeing different types of COVID-19-related content, we created a dummy variable for having shared any of these types of content on social media and a summary variable for the number of types of content shared. We also report findings about some of the individual items in the text, but due to space constraints do not report them in the tables.

To measure more interactive social media engagement, we asked the following concerning social media platforms respondents had indicated using: “Have you had any of the following types of interactions about the Coronavirus pandemic on the social media platforms listed below?” These were the activities: Asked a question about it; Received an answer to a question you asked about it; Answered someone else’s question about it; Participated in a discussion about it; Posted about your own experiences related to it; Asked for support; Offered support; Received support; and Corrected someone else’s post or comment about it. The first five of these we recoded into a dummy variable measuring social media interactions about the pandemic. The three questions about support we recoded into a support-interaction dummy variable. The last one constitutes a variable in its own right about making corrections.

### Analytical Strategy

We start by looking at how people with and without disabilities compare regarding their personal experiences with COVID-19 to establish how the pandemic has affected the two groups. To explore different levels of engagement on social media, we report on both bivariate and multivariate analyses. For the latter, we first rely on logistic regression to look at any exposure or engagement with COVID-19 content on social media, then next we use ordinary least squares (OLS) regression to look at what explains the variation in the number of types of such content people saw, shared, and interacted about. We first run models with disability variables, then investigate whether the relationships change after including health-related variables.

## Findings

### Personal Experiences With COVID-19

**Figure 1.**
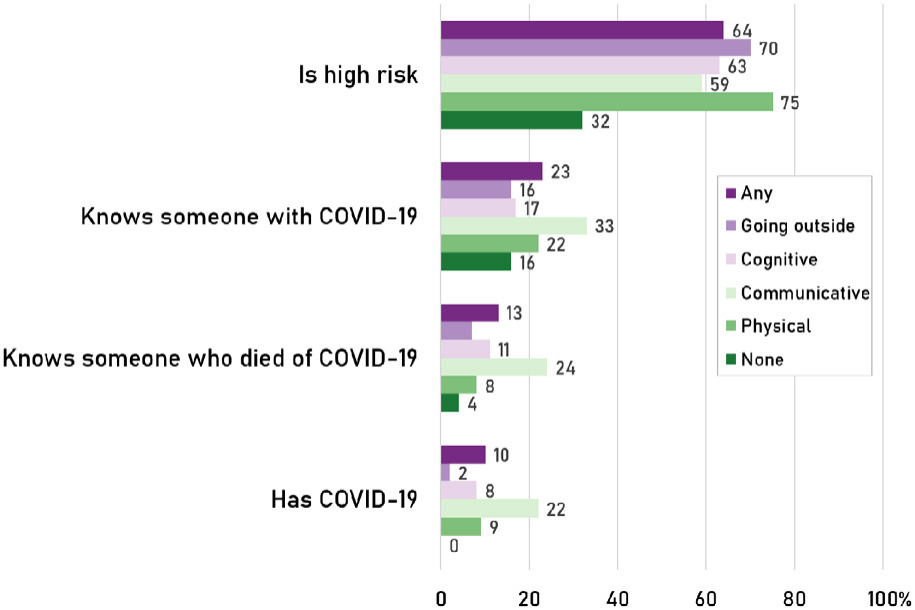
Experiences with COVID-19 by disability status.

PWD reported COVID-19 as featuring much more prominently in their personal lives and social circles than people without disabilities. Among PWD, 64% reported health conditions that put them in the medically high-risk category, a significant contrast to under a third of those without disabilities. Figure 1 breaks down the prevalence by type of disability. One in 10 PWD in our sample reported having been diagnosed with COVID-19 compared with just *one case* among the 1,160 participants without impairments. PWD are also more likely to know others with the COVID-19: 23% of PWD said they knew someone in contrast to 16% of those without. PWD are also much more likely to know people who have died from the disease at 13% as opposed to 4% of those without disabilities.

### Seeing COVID-19-Related Content on Social Media

Four-fifth of respondents reported having seen some type of COVID-19-related content we asked about on social media. The difference between PWD and others is statistically significant with 86% of the former compared with 79% of the latter reporting this. Of the eight types of information, the average PWD reported seeing 5.5 compared with 4.8 among people without disabilities, also a statistically significant difference. Those with a communicative disability and those with cognitive impairment saw the most types, both averaging 5.8 types of information.

To see whether these differences hold once we control for various background characteristics, we ran a logistic regression on having seen any such content by any disability as well as by disability type (Table 1). PWD have higher odds of having seen COVID-19 information on social media than people without disabilities when controlling for sociodemographics and internet experiences and skills. Among disability types, only those with communicative disability have marginally significantly increased odds. When we introduce health-related variables and COVID-19 experience, the relationships hold for any disability, but the relationship with communicative disabilities becomes insignificant.

**Table 1.** Logistic Regression on Seeing Any Information About the Novel Coronavirus on Social Media.

	Seeing coronavirus content on social media											
Age	0.96*** (0.00)	0.96*** (0.01)	0.96*** (0.00)	0.96*** (0.01)	0.96*** (0.00)	0.96*** (0.01)	0.96*** (0.00)	0.96*** (0.01)	0.96*** (0.00)	0.96*** (0.01)	0.96*** (0.00)	0.96*** (0.01)
Female	2.07*** (0.31)	2.04*** (0.31)	2.10*** (0.32)	2.06*** (0.31)	2.12*** (0.32)	2.08*** (0.31)	2.14*** (0.32)	2.10*** (0.32)	2.11*** (0.32)	2.07*** (0.31)	2.11*** (0.32)	2.07*** (0.31)
Education: ≤High school	1.00 (0.19)	1.06 (0.20)	1.01 (0.19)	1.06 (0.21)	1.02 (0.19)	1.06 (0.20)	1.00 (0.19)	1.05 (0.20)	1.00 (0.19)	1.05 (0.20)	1.01 (0.19)	1.06 (0.20)
Education: Some college	1.23 (0.27)	1.24 (0.28)	1.24 (0.27)	1.25 (0.28)	1.25 (0.28)	1.25 (0.28)	1.24 (0.27)	1.25 (0.28)	1.26 (0.28)	1.26 (0.28)	1.26 (0.28)	1.26 (0.28)
Hispanic	1.43 (0.34)	1.36 (0.32)	1.43 (0.34)	1.35 (0.32)	1.43 (0.34)	1.35 (0.32)	1.45 (0.34)	1.37 (0.32)	1.44 (0.34)	1.36 (0.32)	1.43 (0.34)	1.35 (0.32)
Black	1.29 (0.32)	1.24 (0.31)	1.27 (0.31)	1.21 (0.30)	1.29 (0.32)	1.23 (0.30)	1.30 (0.32)	1.24 (0.31)	1.31 (0.32)	1.24 (0.31)	1.28 (0.32)	1.22 (0.30)
Asian	1.08 (0.39)	1.08 (0.39)	1.05 (0.38)	1.05 (0.38)	1.05 (0.38)	1.06 (0.38)	1.07 (0.39)	1.08 (0.39)	1.05 (0.38)	1.05 (0.38)	1.04 (0.38)	1.05 (0.38)
Native American	3.21 (2.42)	2.86 (2.16)	3.21 (2.42)	2.81 (2.12)	3.41 (2.59)	2.95 (2.23)	3.42 (2.59)	2.94 (2.22)	3.35 (2.54)	2.90 (2.20)	3.36 (2.54)	2.88 (2.18)
Household income (log)	1.09 (0.09)	1.04 (0.09)	1.08 (0.09)	1.03 (0.09)	1.08 (0.09)	1.03 (0.09)	1.08 (0.09)	1.03 (0.09)	1.09 (0.09)	1.04 (0.09)	1.07 (0.09)	1.03 (0.09)
Infrequent user	0.69 (0.16)	0.70 (0.16)	0.70 (0.16)	0.71 (0.16)	0.69 (0.16)	0.70 (0.16)	0.71 (0.16)	0.72 (0.16)	0.71 (0.16)	0.72 (0.17)	0.70 (0.16)	0.71 (0.16)
Mobile only home	0.76 (0.21)	0.80 (0.22)	0.78 (0.21)	0.82 (0.23)	0.76 (0.21)	0.80 (0.22)	0.78 (0.21)	0.82 (0.23)	0.78 (0.21)	0.81 (0.22)	0.78 (0.21)	0.82 (0.23)
Internet skills	1.22** (0.08)	1.22** (0.08)	1.22** (0.08)	1.23** (0.08)	1.22** (0.08)	1.22** (0.08)	1.22** (0.08)	1.23** (0.08)	1.22** (0.08)	1.22** (0.08)	1.22** (0.08)	1.22** (0.08)
Health status		0.67^†^ (0.14)		0.69^†^ (0.14)		0.72 (0.15)		0.71^†^ (0.15)		0.69^†^ (0.14)		0.73 (0.15)
High risk		1.46* (0.24)		1.52* (0.25)		1.51* (0.25)		1.52* (0.25)		1.54** (0.26)		1.54* (0.26)
COVID experience		1.61* (0.32)		1.65* (0.32)		1.63* (0.32)		1.65* (0.33)		1.65* (0.33)		1.66* (0.33)
Disability	1.80* (0.42)	1.71* (0.41)										
Physical disability			1.42 (0.39)	1.37 (0.40)								
Communicative disabilities					1.20^†^ (0.93)	1.92 (0.83)						
Cognitive disabilities							2.22 (1.09)	2.10 (1.05)				
Outside disabilities									2.17 (1.11)	2.24 (1.17)		
Multiple disabilities											1.09 (0.33)	1.00 (0.33)
Constant	4.08 (4.15)	5.82^†^ (603)	4.62 (4.67)	6.58^†^ (6.81)	4.53 (4.60)	6.31^†^ (6.54)	4.27 (4.35)	5.99^†^ (6.23)	4.40 (4.47)	6.28^†^ (6.51)	5.00 (5.08)	6.90^†^ (7.15)
*N*	1,354	1,353	1,357	1,356	1,357	1,356	1,354	1,353	1,354	1,353	1,354	1,353
Pseudo-*R*^2^	.12	.13	.11	.12	.11	.12	.11	.12	.11	.12	.11	.12

† *p* < .1. *p < .05. **p < .01. ***p < .001.

To test whether the findings about the number of types of content seen hold when controlling for other factors, we report on the results of OLS regressions on the summary variable by any disability and disability type (Table 2). When controlling for sociodemographic factors as well as internet experiences and skills, we find that PWD saw more types of COVID-19-related information on social media than those without disabilities. Those with a communicative disability were especially likely to see more such content. Of the eight topics we inquired about people seeing on social media, the only one where there was no difference by disability status concerned information about symptoms (results of analyses by social media content type not shown due to space constraints). When we also control for health-related variables and experience with COVID-19, we find that having a disability still makes a difference, and the relationship for those with communicative conditions is reduced, but remains marginally significant.

**Table 2.** OLS Regression on Number of Types of Information About the Novel Coronavirus Seen on Social Media.

	Number of types of novel coronavirus content seen on social media											
Age	−0.06*** (0.01)	−0.07*** (0.01)	−0.06*** (0.01)	−0.07*** (0.01)	−0.06*** (0.01)	−0.07*** (0.01)	−0.06*** (0.01)	−0.07*** (0.01)	−0.06*** (0.01)	−0.07*** (0.01)	−0.06*** (0.01)	−0.07*** (0.01)
Female	0.79*** (0.16)	0.76*** (0.16)	0.81*** (0.16)	0.78*** (0.16)	0.82*** (0.16)	0.78*** (0.16)	0.82*** (0.16)	0.78*** (0.16)	0.81*** (0.16)	0.77*** (0.16)	0.81*** (0.16)	0.77*** (0.16)
Education: ≤High school	−0.19 (0.20)	−0.14 (0.20)	−0.18 (0.20)	−0.13 (0.20)	−0.16 (0.20)	−0.12 (0.20)	−0.19 (0.20)	−0.14 (0.20)	−0.18 (0.20)	−0.14 (0.20)	−0.17 (0.20)	−0.13 (0.20)
Education: Some college	0.19 (0.23)	0.20 (0.23)	0.20 (0.23)	0.21 (0.23)	0.21 (0.22)	0.21 (0.23)	0.20 (0.23)	0.21 (0.23)	0.21 (0.23)	0.21 (0.23)	0.23 (0.23)	0.23 (0.23)
Hispanic	0.35 (0.22)	0.28 (0.22)	0.34 (0.22)	0.27 (0.22)	0.35 (0.22)	0.28 (0.22)	0.36 (0.22)	0.28 (0.22)	0.35 (0.22)	0.28 (0.22)	0.34 (0.22)	0.27 (0.22)
Black	0.08 (0.24)	0.02 (0.24)	0.05 (0.24)	−0.00 (0.24)	0.08 (0.24)	0.02 (0.24)	0.08 (0.24)	0.01 (0.24)	0.08 (0.24)	0.02 (0.24)	0.07 (0.24)	0.01 (0.24)
Asian	0.01 (0.36)	0.06 (0.36)	−0.04 (0.36)	0.02 (0.36)	−0.01 (0.36)	0.04 (0.36)	−0.02 (0.36)	0.04 (0.36)	−0.04 (0.36)	0.02 (0.36)	−0.04 (0.36)	0.02 (0.36)
Native American	0.57 (0.54)	0.43 (0.54)	0.56 (0.54)	0.41 (0.54)	0.61 (0.54)	0.45 (0.54)	0.60 (0.54)	0.43 (0.54)	0.57 (0.54)	0.40 (0.54)	0.59 (0.54)	0.42 (0.54)
Household income (log)	0.12 (0.09)	0.08 (0.09)	0.11 (0.09)	0.08 (0.09)	0.11 (0.09)	0.08 (0.09)	0.12 (0.09)	0.07 (0.09)	0.12 (0.09)	0.08 (0.09)	0.11 (0.09)	0.07 (0.09)
Infrequent user	−0.54** (0.26)	−0.50^†^ (0.26)	−0.52** (0.26)	−0.48^†^ (0.26)	−0.53** (0.26)	−0.50^†^ (0.26)	−0.50^†^ (0.26)	−0.47^†^ (0.26)	−0.49^†^ (0.26)	−0.46^†^ (0.26)	−0.50^†^ (0.26)	−0.47^†^ (0.26)
Mobile only home	0.08 (0.31)	0.13 (0.31)	0.11 (0.31)	0.16 (0.31)	0.06 (0.31)	0.13 (0.31)	0.11 (0.31)	0.17 (0.31)	0.11 (0.31)	0.16 (0.31)	0.10 (0.31)	0.17 (0.31)
Internet skills	0.35*** (0.07)	0.35*** (0.07)	0.36*** (0.07)	0.36*** (0.07)	0.36*** (0.07)	0.36*** (0.07)	0.36*** (0.07)	0.36*** (0.07)	0.36*** (0.07)	0.36*** (0.07)	0.36*** (0.07)	0.36*** (0.07)
Health status		−0.38 (0.23)		−0.36 (0.23)		−0.31 (0.23)		−.34 (0.23)		−0.35 (0.23)		−0.30 (0.23)
High risk		0.49** (0.18)		0.57** (0.17)		0.55** (0.17)		0.58** (0.17)		0.59** (0.17)		0.59** (0.17)
COVID experience		0.38** (0.18)		0.42** (0.18)		0.39** (0.18)		0.44** (0.18)		0.43** (0.18)		0.43** (0.18)
Disability	0.66** (0.21)	0.54** (0.22)										
Physical disability			0.39 (0.28)	0.26 (0.29)								
Communicative disabilities					0.88** (0.34)	0.64^†^ (0.34)						
Cognitive disabilities							0.55 (0.37)	0.42 (0.38)				
Outside disabilities									0.60 (0.44)	0.49 (0.44)		
Multiple disabilities											0.08 (0.31)	−0.10 (0.32)
Constant	4.82*** (1.07)	5.11*** (1.07)	4.95*** (1.06)	5.22*** (1.07)	4.87*** (1.06)	5.14*** (1.07)	4.88*** (1.06)	5.18*** (1.08)	4.88*** (1.07)	5.19*** (1.08)	5.03*** (1.07)	5.27*** (1.07)
*N*	1,354	1,353	1,357	1,356	1,357	1,356	1,354	1,353	1,354	1,353	1,354	1,353
*R* ^2^	.17	.17	.17	.18	.17	.18	.17	.18	.17	.18	.17	.18

*Note*. OLS = ordinary least squares.

† *p* < .1. **p < .05. **p < .01. ***p < .001.

### Sharing COVID-19-Related Content on Social Media

Next, we turn from the relatively passive form of social media participation, seeing content about COVID-19, to a more active form: sharing content with others. Consistent with previous research demonstrating more active online engagement by PWD in online interaction and community (Dobransky & Hargittai, 2016; Ellcessor, 2016), PWD were much more likely at 60% to share COVID-19-related content than people without disabilities at 42%. The only specific type of disability where this finding does not hold is people who have difficulty going outside. These differences are robust to holding sociodemographics and internet experiences constant except that people with cognitive impairment are no longer different from others (Table 3). The odds ratio for sharing COVID-19 information was much higher than the odds ratio for seeing that information. Indeed, the smallest effect—for sharing information regarding symptoms of COVID-19—had PWD at over double the odds of sharing the information as people without disabilities. The largest effect—for people sharing religious sentiments about the virus—revealed three times the odds. The results regarding sharing any COVID-19-related information are robust when we control for health variables and COVID-19 experience. Having fair or poor health is itself *negatively* related to sharing, demonstrating it is a distinct measure from disability status.

**Table 3.** Logistic Regression on Sharing Any Information About the Novel Coronavirus on Social Media.

	Sharing any information about the novel coronavirus on social media											
Age	0.97*** (0.00)	0.97*** (0.00)	0.97*** (0.00)	0.97*** (0.00)	0.97*** (0.00)	0.97*** (0.00)	0.97*** (0.00)	0.97*** (0.00)	0.97*** (0.00)	0.97*** (0.00)	0.97*** (0.00)	0.97*** (0.00)
Female	1.23^†^ (0.14)	1.20 (0.14)	1.24^†^ (0.15)	1.21 (0.14)	1.27* (0.15)	1.23^†^ (0.15)	1.27* (0.15)	1.22^†^ (0.14)	1.25^†^ (0.15)	1.21 (0.14)	1.26^†^ (0.15)	1.22^†^ (0.14)
Education: ≤High school	0.99 (0.15)	1.04 (0.16)	0.99 (0.15)	1.05 (0.16)	1.01 (0.15)	1.06 (0.16)	1.00 (0.15)	1.05 (0.16)	0.99 (0.15)	1.05 (0.16)	1.01 (0.15)	1.06 (0.16)
Education: Some college	1.22 (0.21)	1.25 (0.21)	1.21 (0.20)	1.24 (0.21)	1.27 (0.21)	1.27 (0.22)	1.26 (0.21)	1.27 (0.22)	1.26 (0.21)	1.27 (0.22)	1.25 (0.21)	1.27 (0.22)
Hispanic	1.23 (0.21)	1.15 (0.20)	1.20 (0.20)	1.12 (0.19)	1.22 (0.20)	1.14 (0.19)	1.22 (0.20)	1.13 (0.19)	1.22 (0.20)	1.13 (0.19)	1.21 (0.20)	1.13 (0.19)
Black	1.19 (0.21)	1.14 (0.21)	1.13 (0.20)	1.08 (0.20)	1.19 (0.21)	1.13 (0.21)	1.18 (0.21)	1.12 (0.20)	1.19 (0.21)	1.13 (0.20)	1.16 (0.21)	1.11 (0.20)
Asian	0.95 (0.25)	0.95 (0.26)	0.90 (0.24)	0.90 (0.24)	0.92 (0.24)	0.92 (0.25)	0.89 (0.23)	0.90 (0.24)	0.89 (0.23)	0.90 (0.24)	0.90 (0.24)	0.90 (0.24)
Native American	2.02^†^ (0.86)	1.84 (0.79)	1.93 (0.82)	1.75 (0.75)	2.12^†^ (0.90)	1.89 (0.80)	2.06^†^ (0.87)	1.80 (0.77)	2.03^†^ (0.86)	1.79 (0.77)	2.05^†^ (0.87)	1.83 (0.78)
Household income (log)	1.04 (0.07)	0.99 (0.07)	1.03 (0.07)	0.98 (0.07)	1.02 (0.07)	0.98 (0.07)	1.02 (0.07)	0.97 (0.07)	1.02 (0.07)	0.98 (0.07)	1.02 (0.07)	0.97 (0.07)
Infrequent user	1.16 (0.23)	1.19 (0.24)	1.19 (0.24)	1.21 (0.24)	1.18 (0.23)	1.20 (0.24)	1.22 (0.24)	1.24 (0.24)	1.24 (0.24)	1.25 (0.25)	1.23 (0.24)	1.25 (0.25)
Mobile only home	0.91 (0.21)	0.97 (0.23)	0.96 (0.22)	1.02 (0.24)	0.90 (0.21)	0.97 (0.23)	0.96 (0.22)	1.02 (0.24)	0.96 (0.22)	1.02 (0.24)	0.94 (0.22)	1.00 (0.23)
Internet skills	1.23*** (0.07)	1.23*** (0.07)	1.24*** (0.07)	1.24*** (0.07)	1.24*** (0.07)	1.24*** (0.07)	1.23*** (0.07)	1.24*** (0.07)	1.23*** (0.07)	1.23*** (0.07)	1.23*** (0.07)	1.24*** (0.07)
Health status		0.64* (0.11)		0.62** (0.11)		0.74^†^ (0.13)		0.74^†^ (0.13)		0.71^†^ (0.12)		0.68* (0.12)
High risk		1.37* (0.19)		1.43** (0.19)		1.48** (0.20)		1.55** (0.21)		1.54** (0.20)		1.49** (0.20)
COVID experience		1.62** (0.22)		1.67*** (0.23)		1.64*** (0.23)		1.75*** (0.24)		1.74*** (0.24)		1.67*** (0.23)
Disability	2.71*** (0.47)	2.59*** (0.47)										
Physical disability			3.16*** (0.72)	3.16*** (0.76)								
Communicative disabilities					3.70*** (1.13)	3.15*** (0.99)						
Cognitive disabilities							1.16 (0.33)	1.07 (0.31)				
Outside disabilities									1.79^†^ (0.61)	1.72 (0.61)		
Multiple disabilities											2.21** (0.55)	2.03** (0.53)
Constant	0.88 (0.70)	1.22 (1.00)	1.14 (0.90)	1.60 (1.29)	1.08 (0.85)	1.42 (1.15)	1.23 (0.97)	1.69 (1.36)	1.16 (0.91)	1.60 (1.29)	1.18 (0.93)	1.58 (1.28)
*N*	1,354	1,353	1,357	1,356	1,357	1,356	1,354	1,353	1,354	1,353	1,354	1,353
Pseudo-*R*^2^	.08	.09	.07	.09	.07	.08	.06	.07	.06	.07	.06	.08

† *p* < .1. *p < .05. **p < .01 ***p < .001.

We also ran OLS regressions on the summary sharing variable by both disability and disability type (Table 4). With sociodemographics and internet skills and experiences held constant, PWD shared more types of COVID-19-related information than those without disabilities. The only type of disability for which this was not the case was cognitive impairment. When we introduce health variables and COVID-19 experiences, the results are robust, with the exception of the results for those with difficulty going outside becoming only marginally significant. Once again, the magnitude of effect is much larger (nearly three times as large) for PWD sharing information than it is for the more passive act of simply seeing information.

**Table 4.** OLS Regression on Number of Types of Information About the Novel Coronavirus Shared on Social Media.

	Number of types of information about the novel coronavirus shared on social media											
Age	−0.05*** (0.01)	−0.05*** (0.01)	−0.05*** (0.01)	−0.05*** (0.01)	−0.05*** (0.01)	−0.05*** (0.01)	−0.05*** (0.01)	−0.05*** (0.01)	−0.05*** (0.01)	−0.05*** (0.01)	−0.05*** (0.01)	−0.05*** (0.01)
Female	−0.07 (0.16)	−0.10 (0.16)	−0.04 (0.16)	−0.08 (0.16)	0.00 (0.16)	−0.05 (0.16)	−0.01 (0.17)	−0.07 (0.17)	−0.03 (0.17)	−0.08 (0.17)	−0.03 (0.17)	−0.07 (0.16)
Education: ≤High school	−0.29 (0.21)	−0.21 (0.21)	−0.29 (0.21)	−0.20 (0.21)	−0.24 (0.21)	−0.18 (0.21)	−0.27 (0.22)	−0.19 (0.21)	−0.27 (0.21)	−0.19 (0.21)	−0.26 (0.21)	−0.18 (0.21)
Education: Some college	−0.02 (0.24)	−0.01 (0.24)	−0.03 (0.24)	−0.02 (0.24)	0.06 (0.24)	0.04 (0.24)	0.05 (0.24)	0.04 (0.24)	0.06 (0.24)	0.04 (0.24)	0.03 (0.24)	0.03 (0.24)
Hispanic	0.23 (0.23)	0.12 (0.23)	0.18 (0.23)	0.07 (0.23)	0.21 (0.24)	0.10 (0.23)	0.21 (0.24)	0.08 (0.24)	0.20 (0.24)	0.08 (0.24)	0.21 (0.24)	0.10 (0.24)
Black	0.41 (0.25)	0.33 (0.25)	0.32 (0.25)	0.24 (0.25)	0.41 (0.25)	0.32 (0.25)	0.40 (0.26)	0.30 (0.25)	0.41 (0.26)	0.31 (0.25)	0.38 (0.25)	0.29 (0.25)
Asian	−0.19 (0.38)	−0.18 (0.38)	−0.29 (0.38)	−0.26 (0.38)	−0.24 (0.38)	−0.24 (0.38)	−0.29 (0.39)	−0.28 (0.38)	−0.31 (0.38)	−0.29 (0.38)	−0.28 (0.38)	−0.26 (0.38)
Native American	0.25 (0.57)	0.03 (0.56)	0.18 (0.57)	−0.05 (0.56)	0.35 (0.57)	0.09 (0.57)	0.31 (0.58)	0.03 (0.57)	0.27 (0.58)	0.00 (0.57)	0.30 (0.57)	0.05 (0.57)
Household income (log)	0.02 (0.09)	−0.06 (0.09)	−0.00 (0.09)	−0.09 (0.09)	−0.01 (0.09)	−0.08 (0.09)	−0.01 (0.09)	−0.09 (0.09)	0.01 (0.09)	−0.08 (0.09)	0.02 (0.09)	−0.09 (0.09)
Infrequent user	0.43 (0.28)	0.46^†^ (0.27)	0.45 (0.28)	0.48^†^ (0.27)	0.47^†^ (0.28)	0.49^†^ (0.28)	0.53^†^ (0.28)	0.54^†^ (0.28)	0.55* (0.28)	0.55* (0.28)	0.54^†^ (0.28)	0.55* (0.28)
Mobile only home	0.27 (0.33)	0.37 (0.33)	0.34 (0.33)	0.44 (0.33)	0.24 (0.33)	0.35 (0.33)	0.34 (0.33)	0.44 (0.33)	0.33 (0.33)	0.44 (0.33)	0.30 (0.33)	0.40 (0.33)
Internet skills	0.36*** (0.08)	0.36*** (0.08)	0.38*** (0.08)	0.37*** (0.07)	0.38*** (0.08)	0.37*** (0.08)	0.37*** (0.08)	0.37*** (0.08)	0.37*** (0.08)	0.37*** (0.08)	0.37*** (0.08)	0.37*** (0.08)
Health status		−0.76** (0.24)		−0.84** (0.24)		−0.57* (0.24)		−0.61* (0.24)		−0.65** (0.24)		−0.72** (0.24)
High risk		0.68*** (0.18)		0.74*** (0.18)		0.82*** (0.18)		0.90*** (0.18)		0.89*** (0.18)		0.81*** (0.18)
COVID experience		0.64** (0.19)		0.67*** (0.19)		0.67** (0.19)		0.77*** (0.19)		0.76*** (0.19)		0.68*** (0.19)
Disability	1.53*** (0.22)	1.37*** (0.23)										
Physical disability			1.88*** (0.29)	1.77*** (0.30)								
Communicative disabilities					1.84*** (0.36)	1.47*** (0.36)						
Cognitive disabilities							0.48 (0.40)	0.30 (0.40)				
Outside disabilities									0.99* (0.47)	0.86^†^ (0.47)		
Multiple disabilities											1.42*** (0.33)	1.20*** (0.34)
Constant	3.16** (1.12)	3.78** (1.12)	3.58** (1.12)	4.22*** (1.12)	3.41** (1.12)	3.97*** (1.13)	3.54** (1.14)	4.15*** (1.14)	3.47** (1.14)	4.09*** (1.13)	3.63** (1.13)	4.20*** (1.13)
*N*	1,354	1,353	1,357	1,356	1,357	1,356	1,354	1,353	1,354	1,353	1,354	1,353
*R* ^2^	.12	.15	.12	.15	.12	.14	.10	.13	.10	.13	.11	.14

*Note*. OLS = ordinary least squares.

† *p* < .1. *p < .05. **p < .01. ***p < .001.

### Actively Engaging With Others About COVID-19-Related Content on Social Media

The most active types of online engagement we measured concerned interactions with others about COVID-19 on social media, broken down by discussions, support, and correction of content others shared. In all cases, we find that PWD are much more active than those without disabilities. While over half (54%) of PWD have asked or answered questions or discussed COVID-19 on social media, the figure is just over a third (37%) for people without disabilities. We see similarly stark contrasts for support messages (47% vs. 30%, *p* < .001) and correcting someone else’s post or comment about it (37% vs, 22%, *p* < .001). As with the other activities, these are mainly the case for those with physical or communicative disabilities and less so for those with cognitive impairments or difficulty going outside. All of these results are robust when we control for other factors in the regression analyses, including health variables and COVID-19 experience. Table 5 displays the results for disability with controls for other factors.

**Table 5.** Logistic Regression on Various Types of Active Engagement With Others About the Novel Coronavirus on Social Media.

	Interacting with others about the novel coronavirus on social media						Interacting about support around the novel coronavirus on social media						Correcting information posted by someone else about the novel coronavirus on social media					
Age	0.96*** (0.00)	0.96*** (0.00)	0.96*** (0.00)	0.96*** (0.00)	0.96*** (0.00)	0.96*** (0.00)	0.96*** (0.00)	0.96*** (0.00)	0.96*** (0.00)	0.96*** (0.00)	0.96*** (0.00)	0.96*** (0.00)	0.96*** (0.01)	0.96*** (0.01)	0.96*** (0.01)	0.96*** (0.01)	0.96*** (0.01)	0.96*** (0.01)
Female	0.70** (0.09)	0.71** (0.09)	0.72** (0.09)	0.72** (0.09)	0.72** (0.09)	0.71* (0.09)	0.93 (0.12)	0.94 (0.12)	0.95 (0.12)	0.94 (0.12)	0.94 (0.12)	0.94 (0.12)	0.59*** (0.08)	0.60*** (0.08)	0.61*** (0.09)	0.60*** (0.08)	0.60*** (0.08)	0.60*** (0.08)
Education: ≤High school	0.71* (0.11)	0.71* (0.11)	0.72* (0.12)	0.71* (0.11)	0.71* (0.11)	0.72* (0.12)	0.64** (0.11)	0.64** (0.11)	0.66* (0.11)	0.66* (0.11)	0.66* (0.11)	0.66* (0.11)	0.71^†^ (0.13)	0.71^†^ (0.13)	0.73^†^ (0.13)	0.73^†^ (0.13)	0.73^†^ (0.13)	0.73^†^ (0.13)
Education: Some college	0.67* (0.12)	0.67* (0.12)	0.68* (0.12)	0.69* (0.12)	0.69* (0.12)	0.69* (0.12)	0.74^†^ (0.13)	0.73^†^ (0.13)	0.76 (0.14)	0.77 (0.14)	0.77 (0.14)	0.76 (0.14)	0.81 (0.16)	0.80 (0.16)	0.83 (0.16)	0.82 (0.16)	0.82 (0.16)	0.82 (0.16)
Hispanic	1.11 (0.19)	1.08 (0.19)	1.10 (0.19)	1.09^†^ (0.19)	1.08 (0.19)	1.09 (0.19)	1.37^†^ (0.24)	1.34^†^ (0.23)	1.36^†^ (0.24)	1.33 (0.23)	1.33 (0.23)	1.34^†^ (0.23)	1.20 (0.23)	1.16 (0.22)	1.19 (0.23)	1.19 (0.22)	1.18 (0.22)	1.18 (0.22)
Black	1.43^†^ (0.26)	1.37^†^ (0.25)	1.42^†^ (0.26)	1.39^†^ (0.26)	1.39^†^ (0.26)	1.39^†^ (0.26)	1.11 (0.21)	1.04 (0.20)	1.10 (0.21)	1.08 (0.20)	1.08 (0.20)	1.08 (0.20)	1.32 (0.26)	1.24 (0.25)	1.31 (0.26)	1.31 (0.26)	1.31 (0.26)	1.28 (0.25)
Asian	0.77(0.22)	0.73 (0.21)	0.75 (0.21)	0.73 (0.21)	0.72 (0.20)	0.74 (0.21)	0.86 (0.25)	0.82 (0.24)	0.84 (0.24)	0.80 (0.23)	0.80 (0.23)	0.83 (0.24)	0.81 (0.26)	0.78 (0.25)	0.79 (0.26)	0.77 (0.25)	0.77 (0.25)	0.79 (0.25)
Native American	1.32 (0.61)	1.26 (0.52)	1.36 (0.57)	1.29 (0.54)	1.27 (0.53)	1.31 (0.55)	0.50 (0.25)	0.48 (0.23)	0.53 (0.26)	0.50 (0.25)	0.50 (0.24)	0.51 (0.25)	0.96 (0.46)	0.92 (0.44)	1.00 (0.48)	0.96 (0.46)	0.96 (0.46)	0.96 (0.46)
Household income (log)	1.00 (0.07)	0.99 (0.07)	0.99 (0.07)	0.98 (0.07)	0.98 (0.07)	0.98 (0.07)	1.01 (0.07)	0.99 (0.07)	1.01 (0.07)	0.99 (0.07)	0.99 (0.07)	0.99 (0.07)	1.02 (0.08)	1.00 (0.08)	1.01 (0.08)	1.01 (0.08)	1.01 (0.08)	1.00 (0.08)
Infrequent user	1.25 (0.27)	1.26 (0.27)	1.27 (0.27)	1.31 (0.28)	1.31 (0.28)	1.31 (0.28)	1.48^†^ (0.32)	1.49^†^ (0.32)	1.51^†^ (0.32)	1.55* (0.33)	1.55* (0.33)	1.57* (0.33)	1.44 (0.34)	1.44 (0.34)	1.48^†^ (0.35)	1.55^†^ (0.36)	1.55^†^ (0.36)	1.55^†^ (0.36)
Mobile only home	0.95 (0.24)	1.00 (0.25)	0.93 (0.24)	1.00 (0.25)	1.00 (0.25)	0.98 (0.25)	1.15 (0.30)	1.21 (0.31)	1.13 (0.29)	1.21 (0.31)	1.21 (0.31)	1.18 (0.30)	1.35 (0.37)	1.42 (0.39)	1.30 (0.37)	1.44 (0.39)	1.44 (0.40)	1.39 (0.38)
Internet skills	1.26 *** (0.07)	1.27*** (0.07)	1.27*** (0.07)	1.27*** (0.07)	1.27*** (0.07)	1.26*** (0.07)	1.20** (0.07)	1.22** (0.07)	.155** (0.07)	1.22** (0.07)	1.22** (0.07)	1.21** (0.07)	1.34*** (0.09)	1.36*** (0.09)	1.36*** (0.09)	1.37*** (0.09)	1.37*** (0.09)	1.36*** (0.09)
Health status	0.70^†^ (0.13)	0.69^†^ (0.13)	0.77 (0.14)	0.75 (0.14)	0.74 (0.14)	0.72^†^ (0.14)	0.64* (0.13)	0.60* (0.12)	0.71^†^ (0.14)	0.70^†^ (0.14)	0.69^†^ (0.14)	0.66* (0.13)	0.75 (0.17)	0.71 (0.16)	0.83 (0.18)	0.81 (0.18)	.82 (0.18)	.75 (0.17)
High risk	1.37* (0.20)	1.44** (0.20)	1.45** (0.21)	1.54** (0.22)	1.54** (0.22)	1.46** (0.21)	1.43* (0.21)	1.49** (0.22)	1.55** (0.22)	1.66*** (0.24)	1.66*** (0.24)	1.55** (0.23)	1.54** (0.25)	1.63** (0.26)	1.66** (0.26)	1.77*** (0.28)	1.79*** (0.28)	1.64** (0.26)
COVID experience	1.67*** (0.24)	1.72*** (0.24)	1.69*** (0.24)	1.79*** (0.25)	1.78_***_ (0.25)	1.71*** (0.24)	1.30^†^ (0.19)	1.33* (0.19)	1.52^†^ (0.19)	1.40*(0.20)	1.40* (0.20)	1.33* (0.19)	1.39* (0.22)	1.43* (0.22)	1.40* (0.22)	1.50** (0.23)	1.50** (0.23)	1.41* (0.22)
Disability	2.00*** (0.36)						2.17*** (0.39)						2.05*** (0.39)					
Physical disability		2.07** (0.49)						2.92*** (0.68)						2.58*** (0.64)				
Communicative disabilities			2.67** (0.78)						2.55** (0.69)						2.72*** (0.76)			
Cognitive disabilities				1.17 (0.34)						1.03 (0.30)						1.15 (0.36)		
Outside disabilities					1.47 (0.52)						1.19 (0.44)						1.00 (0.41)	
Multiple disabilities						1.83* (0.48)						1.97** (0.50)						2.32**(0.62)
Constant	2.18 (1.86)	2.70 (2.29)	2.30 (1.95)	2.81 (2.39)	2.76 (2.34)	2.66 (2.25)	1.15 (1.01)	1.50 (1.31)	1.23 (1.08)	1.42 (1.24)	1.40 (1.22)	1.44 (1.25)	0.60 (0.58)	0.78 (0.75)	0.61 (0.59)	0.62 (0.60)	0.63 (0.61)	0.76 (0.72)
*N*	1,353	1,356	1,356	1,353	1,353	1,353	1,353	1,356	1,356	1,353	1,353	1,353	1,353	1,356	1,356	1,353	1,353	1,353
Pseudo-*R*^2^	.13	.12	.13	.12	.12	.12	.10	.10	.10	.09	.09	.09	.12	.13	.12	.12	.12	.12

† *p* < .1. *p < .05. **p < .01. ***p < .001.

## Discussion and Conclusion

Results based on our national survey administered during the first few weeks of the COVID-19 pandemic in the United States demonstrate the importance of social media in the lives of PWD during this turbulent time. Due to public health measures, the entire country experienced increased limitations on their mobility and activities, putting the internet more front-and-center in daily life than it already was. Although people from all walks of life were locked down, not everyone reacted to the effects of the pandemic uniformly. PWD exceeded those without disabilities in use of social media to keep abreast of information about COVID-19, to share related information, and to engage in a wide array of interactions about it.

Given the overlap between chronic illness and disability (an incomplete overlap), it makes sense that PWD would be more actively engaged with information about an emerging health threat. PWD are commonly more interested in health information online than the general population (Andreassen et al., 2007; Dobransky & Hargittai, 2006; Fox, 2007). Clearly, this pandemic deeply affected the lives of PWD, as they are more likely to know people who have COVID-19 and to have it themselves. However, it is striking that in most cases the results hold even when introducing controls for health status, conditions with elevated risk for negative COVID-19 outcomes, and personal experiences with COVID-19. Active engagement on social media about the pandemic by PWD cannot be explained away as simply an artifact of their own increased risk.

The results confirm previous research on the active nature of PWD online (Dobransky & Hargittai, 2016; Ellcessor, 2016; Trevisan, 2017; van Deursen & van Dijk, 2014), demonstrating that this applies to social media during a health crisis. The results show that with sociodemographics, internet experiences and skills, and health-related variables held constant, PWD as a whole used social media for COVID-19-related information more and reported seeing more kinds of information about COVID-19 than those without disabilities. When we move to more active types of engagement—sharing information about the virus and interactions—on social media, however, we see not only stronger effects but also effects that apply to several—though not all—disability types.

Although our study did not ask questions about usability, the findings about PWD’s active online engagement suggest that tools exist for people with varying needs, preferences, and abilities to navigate digital resources. This points to improvements in access through movements such as universal design and universal usability, and the importance of maintaining and building on these successes (A. Lazar et al., 2016; J. Lazar & Jaeger, 2011). As social media and other digital platforms have become more accessible, PWD have been increasingly able to make use of them for tasks and interactions, both general and disability specific, that enhance their lives. In fighting to have these options available and making use of them for video chatting, working remotely, and activism, for instance, PWD likely paved the way for millions of those who were scrambling to put them to use during the social upheavals of the pandemic. This is a good example of how accommodations for PWD ultimately benefit the population at large. The results show that PWD continued to make more active use of online tools during the pandemic. As society now ponders the possibility of maintaining the wide availability of these options longer-term, the work of PWD in building the foundation of this infrastructure should be noted.

Like all studies, ours has limitations. Because we had no way of oversampling PWD, their numbers are low in some cases (cognitive impairment and difficulty going outside in particular). It may be that some of our nonfindings for those groups are attributable to this. Future work could gather data on a larger sample with the hopes of capturing more PWD to address this limitation. It is also possibly the case that some PWD with more extreme levels of disability may be limited in taking surveys and/or the time needed for it is too precious compared with their limited time on other activities to have participated thereby excluding certain PWD groups from the sample. Despite these issues, we are encouraged by the overall percentage of PWD in the sample and given the rare focus on this population believe that our paper makes an important contribution to the literature about their online experiences, and more generally how people experienced the first weeks of COVID-19 with respect to social media usage.
